# 

**Published:** 1896-10-03

**Authors:** 


### The Hospital

A JOURNAL OF

THE MEDICAL SCIENCES AND HOSPITAL ADMINISTRATION,

INCLUDING

A SPECIAL SECTION FOR NURSES.

EDITED BY

HENRY C. BURDETT,

AND

SOLOMON C. SMITH, M.D.

IN TWO VOLUMES ANNUALLY.

**VOLUME XXI.**

October, 1896, to March, 1897.

SURGEON GEN'L'S OFFICE 158764 LIBRARY.

London:

PUBLISHED BY THE SCIENTIFIC PRESS (LIMITED), AT THE HOSPITAL BUILDING, 28 & 29, SOUTHAMPTON STREET, STRAND, W.C.

MDCCCXCVII.

W1
H069X

### INDEX TO VOL. XXI., 1896–97.

*** Articles under the general heading *Medical Progress and Hospital Clinics* are distinguished by the initial (C.) for *Hospital Clinics*, and by (P.) for the rest of the series. (W.) distinguishes the *Institutional Workshop* articles.

Abdominal Tumours, False (P.), 266
Abdominal Viscera, Diseases of (P.), 265
Abductor Paralysis (P.), 398
Abscess, Cerebral (P.), 148
Abscess, Pulmonary (P.), 129
Acne (P.), 351
Addenbrooke's Hospital (W.), 220
Adelaide Hospital and the Government of South Australia, The (W.), 15
Adenoid Growths (P.), 29
Adenoids (P.), 267
Adulterators and Their Ways, 123
Albuminuria (P.), 7
Albuminuria, Cyclic in a Family (P.), 350
Alcohol (P.), 431
Alcohol and Digestion, 105
Alcohol in Acute Disease, 40
Anastomosis, Intestinal (P.), 217
Anæmia (P.), 380; Pernicious, 381; Splenic, 381
Anæsthetics, The Administration of, 105
Anæsthesia, The Jubilee of (C.), 44
Anæsthetics, The Dangers of, 424
Anæsthetics, Instrumental to the Administration of (P.), 64, 81
Anæsthetics, The Jubilee of, 39
Angina Ludovici (C.), 77
Antiseptics in Lying-in Hospitals (C.), 346
Anti-Vaccination, A New Departure, 3
Anti-Vaccinators' Case in the Report of the Vaccination Commission, 374
Anus, Artificial and Entero-vaginal Fistula (P.), 216
Arabian and Mohammedan Influence, The, 75
Asylum Administration (W.), 353
Arteries, End-to-End Approximation of Cut (C.), 313
Around the Hospitals, 36
B.M.A., The, and Medical Defence, 277
Baby-Farming in France, 279
Bacteriology and State Aid, 175
Barnstaple Infirmary (W.), 186
Bedrooms, The Air of, 209
Birmingham Hospital Saturday Fund (W.), 117
Bladder and Urethra, Rupture of the (P.), 27
Bladder, Ulceration of the (P.), 49
Bone Defects of the Skull, Treatment of (P.), 112
Book World of Medicine, The (*Books, Magazines, and Pamphlets reviewed or noticed and articles relating to*):—
  Allbutt and Playfair. A System of Gynæcology, 273
  Barr. Manual of Diseases of the Ear, 187
  Brodhurst. Observations on Congenital Dislocation of the Hip, 137
  Brown. *See Wright.*
  Buck. A Vest-Pocket Medical Dictionary, 255
  Cautley. Natural and Artificial Methods of Feeding Infants and Young Children, 369
  Cheyne. Objects and Limits of Operations for Cancer, 85
  Coombs. Galvanism in the Treatment of Neuritis and Other Diseases, 19
  Cottage Hospital Case-book, The, 369
  Doyen. Traitement des Suppurations Pelviennes, 155
  Dutton. Obesity, its Causes and Treatment, 19
  Ewart. Gout and Goutiness and their Treatment, 119
  Exposures of Quackery, 401
  Foster. Course of Elementary Practical Physiology and Histology, 369
  Fowler. *See Marshall.*
  Freyer. Modern Treatment of Stone, &c., by Litholapaxy, 37
  Gemmell. Chemical Notes and Equations, 119
  Green. Manual of Botany, 69
  Hall. Aids to Obstetrics, 37
  Hyde. Causes and Treatment of Rheumatoid Arthritis, 85
  Imperial Health Manual of Germany, 53
  Jones. Practical Manual of Diseases of Women and Uterine Therapeutics, 437
  Kelly's London Medical Directory, 1897, 401
  Legge. Public Health in European Capitals, 137
  Maclagan. Rheumatism, 69
  Marshall. The Frog, an Introduction to Anatomy, Histology, and Embryology, 101
  Marson. Charity Organisation and Jesus Christ, 355
  Midwifery (Catechism Series), 221
  Muir. Story of the Chemical Elements, 355
Book World of Medicine (*continued*).
  Paget. Surgery of the Chest, 53
  Peachey. Everybody's Guide to Chess and Draughts, 119
  Playfair. *See Allbutt.*
  Ransome. Treatment of Phthisis, 239
  Richards. Guide to the Examination in Practical Chemistry for the Conjoint Board, 137
  Roberts. Practice of Midwifery, 239
  Saundby. Lectures on Renal and Urinary Diseases, 221
  Smith. Angio-Neurosis: Being Studies in the Diseases of the Vaso-Motor System, 53
  Spinks. Drainage of Villages, 101
  Stedman. Twentieth Century Practice of Modern Medicine, 19, 37, 101, 401
  Stewart. Increase of General Paralysis in England and Wales, 155
  Swain. Surgical Emergencies, 69
  Tebb and Vollum. Premature Burial and How it may be Prevented, 171
  Thudicum. Progress of Modern Chemistry, 355
  Tubby. Deformities: A Treatise on Orthopædic Surgery, 101
  Vollum. *See Tebb.*
  Whitaker's Almanack, 1897, 256
  Wright and Brown. Everybody's Cycling Law, 119
  Year Book of Treatment for 1897, 401
  Year Books, 255
  Yeo. Food in Health and Disease, 85
Bone Defects of the Skull, Treatment of (P.), 112
Bradshaw Lecture on Subjective Sensation of Sound (C.), 109
Brain, Exploration of the (P.), 112
Brain, The Training of the, 275
Brain Tumours (P.), 147
Bridgnorth, New Infirmary at (W.), 35
British Army, The British Parliament and the, 289
Bubonic Plague (C.), 246
Bunions and their Treatment (C.), 363
C.D. Acts, Some Moral Aspects of the, 373
Caged Children and Christian Civilisation, 121
Calculus, Vesical (P.), 49
Cancer of the Tongue, A Clinical Lecture on (C.), 145
Cancer of the Uterus, Diagnosis of (C.), 429
Cancer, Retrogression of. *See p.* 186
Carcinoma of the Stomach (P.), 199
Central Hospital Board Question, The (W.), 303
Cervical Sympathetic, Section of the (P.), 29
Charing Cross Hospital (W.), 169
Charity and State Aid, 175
Chemistry, Real and Examinational, 325
Chilblains, 309
Child Life Under Queen Victoria, 329, 345, 361, 377, 393, 411, 427
Children, Diseases of (P.), 348
Children, Physically and Mentally Defective, 57
Children's Wards. *See p.* 186
Chlorosis (P.), 380
Cholelithiasis (P.), 285
Cholera (P.), 397
Cholera in January, 258
Cirrhosis of the Liver (P.), 350
City of London Hospital for Diseases of the Chest (W.), 370, 403
Clinical Demonstration, A (C.), 179
Clinical Society, The (C.), 46, 76, 126, 162, 194, 262, 346, 378
Colour-blind Sailors and the Board of Trade, 103
Colour-blind Seamen, Our, 391
Commissariat (Hospital Expenditure) (W.), 33, 51, 115, 133, 201, 269, 319, 402
Construction Notes (W.), 35, 153, 186, 238, 289, 305, 421
Contre-coup, The Mechanism of Cerebral Injury by (P.), 96
Convict, The Competition of the, 245
Coroners and Juries, 209
Correspondence. *See Editor's Letter Box*
Cottage Hospital, 151
Cottage Hospitals and Medical Fees, 186, 238
Craniectomy (P.), 112
Crime, Physical Inferiority and, 357
Cure, What is a? 56
Cycle in Spinal Curvature, The, 123
Cyst, Hydatid of the Pleural Cavity (P.), 130
Cystonephrosis (P.), 433
Cysts, Hydatid, of the Liver (P.), 284
Cysts, Pancreatic and Retro-peritoneal (P.), 316
Dangers of Funerals, The, 123
Deaf and Dumb, The Education of (W.), 271
Death Tax upon Poverty, The, 343
Decency in Factories, The Lack of, 41
Delirium, Acute (P.), 232
Derbyshire Royal Infirmary (W.), 272
Dermatology (P.), 298, 317, 352
Diabetes, Etiology, Pathology (P.), 9
Difficulties of Organising the Profession, 104
Disgrace to England, A, 423
Diphtheria (P.), 417
Diphtheria, Schools and, 359, 391.
Diphtheria, The Serum Treatment of, 73, 217
Disease, The Causes of, 277
Disinfection of Ships, The, 259
Disinfection of Rooms (P.), 302
Doctors: Should Doctors be Peers? 71
Doctors and the Peerage, 41
Doctors, Nurses, Patients, and Lady Priestley, 242
Drug: To Drug or not to Drug, 409
Drunken Women, The Prison Treatment of, 409
Dundee Royal Infirmary, Nurses' Home (W.), 67
Dying, The Torture of the, 276
Ear, External, Deformity of the (P.), 350
Ear, Middle, Disease of, and its Sequelæ (P.), 148
Eczema (P.), 298
Edinburgh, New Fever Hospital at (W.), 289
Edinburgh Royal Infirmary, The (W.), 353
Edinburgh Royal Infirmary New Laundry Offices (W.), 305
Editor's Letter-box, 66, 155, 171, 238, 254, 272, 289, 304, 322, 338, 354, 385, 437
1896: A Retrospect, 226
Empyæma, Antral (P.), 267
Empyæma, Double (P.), 130
Empyæmas, Sinus (P.), 267
Enterol (P.), 217
Epidemiological Society (C.), 280, 378
Epilepsy (P.), 232
Epilepsy, Traumatic (P.), 96
Erichsen and Humphry, 1
Erythema Nodosum of the Larynx and Trachea (P.), 399
Eucaine (P.), 368
Exophthalmos (P.), 29
Eye, Injuries of the (C.), 127
Falkirk New District Fever Hospital, 421
False Teeth, The Danger of (C.), 281
Female Mortality in the Liquor Traffic, 225
Ferratin (P.), 200
Fever Hospital at Edinburgh, New (W.), 289
Fevers (P.), 364, 396, 415
Filth and Epidemics, 242
Fire or Frost in Medicine? 408
Flatfoot and Its Treatment (C.), 281, 297
Floor, Treating a Pine Wood. *See p.* 171
Flowers for London Hospitals. *See p.* 171
Flowers in Bedrooms, 89
Food and Evolution, 425
Food, Government of the People's, 59
Foods (P.), 431
Fracture, Ununited, its Causes and Treatment (C.), 230
Fractures and Dislocations (P.), 47
Gastrectasis (P.), 282
Gastric Varix (P.), 282
Gastritis, Phlegmonous (P.), 282
Gastrorraphy (P.), 199
Gastrostomy (P.), 199
Gelanthum (P.), 132
General Medical Council, The, 183
General Medical Council, The Election to the, 209
Genu Valgum Adolescentium (P.), 367
German Bands, The Physiological Action of, 159
Giants and Dwarfs, 89
Glasgow Samaritan Hospital (W.), 158
Gold, The Winning of, 211
Gout (P.), 62, 63
Great Northern Central Hospital, The (W.), 185
Greater Cleanliness of the Body, The, 375
Grubbing and Tippling, 293
Guild of St. Luke, The (W.), 83
Guy's Hospital (W.), 202
Hæmaturia, The Diagnosis of (P.), 432
Halifax Royal Infirmary (W.), 13
Harrogate Cottage Hospital (W.), 272
Harveian Oration, The, 57
Harveian Society, The (C.), 61, 144, 362, 428
Head Injuries (P.), 95

Heart, The Complete Diagnosis of Disease of the (C.), 60
Hellenic and Greek Influence (Sociology), 5
Hepatic Abscess (P.), 284
Heroic Doctors, 359
Hip, Congenital Displacement of the (P.), 367
Hip Joint, Tuberculous Disease of the (C.), 6
Hospital, The, to its Readers, 2
Hospital Administration (W.), 15, 185, 219
Hospital Construction (W.), 13, 67, 99, 151, 287, 403
Hospital Expenditure (W.), 33, 51, 115, 133, 201, 269, 319, 402
Hospital Festivals and Meetings (W.), 203, 321, 338, 354, 370, 386, 404, 421, 435
Hospital Notes (W.), 237, 419
Hospital Nurses in Asylum Service (W.), 354
Hospital Problem and the Prince's Fund, The, 341
Hospital Reform Association, The, 73, 219
Hospital Refuse, The Destruction of (W.), 236
Hospital Saturday Fund and Street Collections (W.), 136
Hospital Ships, 66
Hospitals and Milkmen, 323
Hospitals, Tenders and Contractors (W.), 403
House Divided against Itself, A, 207
House Warranty, 225
Howard Association (W.), 151
Hunger Whip, The, 309
Hutchinson, Mr., and Medical Leadership, 23
Hydatid Cyst of the Pleural Cavity (P.), 130
Hydatid Cysts of the Liver (P.), 284
Hydroceles, Bilocular, Intrapelvic, and Scrotal (P.), 418
Hygienic Aspect of Wood Pavement, The, 23
Hyperostosis Cranii (P.), 111
Hypertrophic Pulmonary Osteo-Arthropathy (P.), 129
Ices in Midwinter, 191
Indian Famine and English Hospitals (W.), 288
Infants, Diseases of, during Lactation (P.), 349
Infectious Diseases arising in Children's Hospitals, The Treatment of, 213
Infectious Diseases in Dublin, 52
Infirm, The Fate of the, 22
Infirmary Question at Manchester, The (W.), 253
Influenza and Insanity, 389
In-growing Toenail (C.), 331
"Ins and Outs," 175
Insanity (P.), 196
Insane. The Story of the Insane from Year to Year, 18, 34, 68, 84, 117, 153, 221, 272, 321, 353
Insanity, The Early Diagnosis of (C.), 93
Insanity following Head Injury (P.), 96
Isolation Hospitals Act. See p. 154
Intestinal Anastomosis (P.), 217
Intestinal Obstruction (P.), 216
Intestine, Tuberculosis of, by Ingestion (P.), 349
Intestines, Diseases of the (P.), 78, 265
Intra-Cranial Operations (P.), 434
Intra-dural Hæmatoma, Lumbar Puncture of (P.), 97
Intra-Peritoneal Injections, Notes on C.), 412
Intussusception (P.), 216
Kidney Diseases (P.), 332
Kidney, Movable (P.), 432
Kidney Tension (P.), 332
Kidney, Traumatic Lesions of the (P.), 432
Kidney, Tuberculosis of the (P.), 432
Kidneys and Ureters, Surgery of the (P.), 432
Klumpke's Paralysis (P.), 249
Labyrinth, Affections of the (P.), 149
Ladies and Bagpipes, 3
Lady Guardian. Is the Lady Guardian a Failure? 105
Landry's Paralysis (P.), 249
Langholm, New Hospieal for (W.), 35
Laparotomy, Weak Scars and Hernia following (P.), 167
Laryngitis, Catarrhal, Influenzal (P), 399
Larynx, Diseases of the (P.), 367, 382, 398, 416; Examination of the, in Young Children (P.), 367; Innervation of the (P.), 367
Lenticular Ganglion, Anatomy of the (P.), 29
Lesions, Acute Pontine (P.), 249
Leucocythæmia (P.), 381
Leukæmic Infiltration (P.), 382
"Levelling up the Villages," 73
Liver, Hydatid Cysts of the (P.), 284; Movable (P.), 285
Livingstone and the Mission of Medicine, 191, 255
Llangammarch Wells as a Health Resort (W.), 17
Local Government, 141
London, A New Danger for, 89
London Hospital, The (W.), 185
London Water Supply and the Experts, 141
London Water Supply, The 87
Loretin (P.), 252
Loughborough Hospital and Dispensary (W.), 186
Lumbar Puncture (P.), 96
Lupus (P.), 298
Malaria (P.), 398
Medical Certificates, 307
Medical Club, a National, 277
Medical Peer, The First, 241
Medical Schools, The Introductory Addresses at the, 25; Teaching in, 41
Medical Society, The (C.), 45, 76, 144, 212, 280, 363, 394, 428
Medical Society of London (C.), 108
Medicine, English, in the Victorian Age, 257
Medico-Legal, 205
Medico-Psychological Association and their Nurses, The, 238, 254, 272, 304, 322, 339
Membraneous Sore Throat (P.), 382
Meningitis in its Surgical Aspect (P.), 150
Meningocele and Spina Bifida (P.), 97
Metropolitan Hospital, The "Censure" on the (W.), 235
Metropolitan Hospital Sunday Fund (W.), 203
Midwives, Registration of. See p. 385
Modern Sociology, 5, 43, 59, 75, 91, 107, 125, 143, 161, 177, 193, 211, 245, 261, 279, 295, 311
Mortuaries, Waiting, 158
Mucous Polypi in the Stomach (P.), 282
Nasal Mucosa, Papillomatous Degeneration of the (P.), 266
Nasal Polypus (P.), 266
Nasal Reflexes (C.), 413
Nasal Stenosis (P.), 234
Nasal Tuberculosis (P.), 266
Nature and Advertisement, 191
Nerve Avulsion, Thiersch's Method of (P.), 433
Nerve Cells, Anatomy and Physiology of (P.), 214
Nerve Section (P.), 433
Nerve Stretching (P.), 433
Nerve Study in a Nervous Age, 139
Nerve Suturing and Nerve Loosening (P.), 29
Nerves, Surgery of the (P.), 27, 433
Nervous System, Diseases of the (P.), 165, 181, 196, 214, 232, 248
Neuralgia, Trifacial (P.), 10, 28, 433
Neurasthenia (P.), 182
Neuroses, Reflex (P.), 31
New Appliances and Things Medical, 11, 31, 50, 98, 114, 132, 150, 168, 184, 200, 218, 252, 268, 286, 302, 318, 368, 384, 400, 418, 434
New York, Paupers and Criminals in, 295
Newport Infectious Diseases Hospital (W)., 35
North Devon Infirmary (W.), 186
Nose, Tuberculosis of the (P.), 30, 266
Nose and Throat, Diseases of, in Relation to Diseases of Digestion, 250
Notes and News, 4, 24, 42, 58, 74, 90, 106, 124, 142, 161, 177, 192, 210, 246, 260, 279, 294, 310, 328, 345, 360, 376, 392, 410, 426
Œsophagus, Foreign Bodies in the, 198
Œsophagus and Stomach, Surgery of the (P.), 198
Ophthalmia in the Army—A Contrast, 308, 339
Ophthalmology, Progress in (P.), 112, 131
Optic Nerves, Decussation of the (P.), 232
Orchectomy, Double, for Prostatic Enlargement (C.), 313
Ormskirk Cottage Hospital (W.), 287, 339, 403
Orthopædic Surgery (P.), 350, 366
Osteo-Arthropathy, Hypertrophic Pulmonary (P.), 129
Otitis, Radical Operations for Chronic Suppurative (P.), 149
Ovariin (P.), 63
Oxygen, Ulcers and Micrococci (C.), 330 and see p. 385
Oxygen in Treatment of Diseases of the Nose (P.), 267
Oxygen Treatment of Ulcers, The, 390 (C.), 395, and see p. 437
Oysters, The Trade in, 174
Ozœna (P.), 31
Ozone as a Therapeutic Agent (P.), 334
Pain, The Riddle of, 157
Pancreas, Diseases of the (P.), 266
Pancreas and Spleen, Surgery of the (P.), 316
Pancreatic and Retro-peritoneal Cysts (P.), 316
Papilloma (P.), 399
Paralysis, Acute Ascending (Landry's) (P.), 249
Paralysis, General (P.), 233
Paralysis, Klumpke's (P.), 249
Parasitic Diseases (P.), 317
Passmore Edwards Convalescent Home (W.), 153
Patient's Consent to Operations, 122
Perforating Ulcer (P.), 216
Peritoneal Adhesions (P.), 167
Peritoneum, Pathology of the (P.), 265
Peritonitis, Acute General (P.), 167
Peritonitis, Tuberculous (P.), 167, 265
Phthisis (P.), 301
Physician, the Hospital, and the Community, The, 190
Physiology and Cold Feet, 293
Pitfalls of Practice, Some, 223
Plague. Could the Plague Attack England? 189
Plague. Is Plague Contagious? 391
Plague, The, 343 (P.), 397
Plague, Bubonic (C.), 246
Plague, London and the, 343
Plague, The Waning of the (C.), 429
Pneumatic Cushions for Sleeping Cars, 159
Pneumonia, The Toxin of, 326
Pontine Lesions, Acute (P.), 249
Poor Law Officers' Superannuation (W.), 204
Practical Departments (W.), 35, 322
Practical Points (W.), 171, 322
Prince of Wales's Hospital Fund for London (W.), 335
Prison Life, Mistaken Impressions of, 91, 107, 125, 161, 177
Prostate, Surgery of the (P.), 383
Prostatic Enlargement, Double Orchectomy for (C.), 313
Psoriasis (C.), 128
Public-House Reform, 309
Puerperal Fever, The Serum Treatment of (C.), 92
Puerperal Fever, On the Undiminished Mortality from, in England and Wales, 379
Puerperal Fever, Further Observations on the Arrest of (C.), 413
Pulmonary Abscess (P.), 129
Pulse, The (C.), 195
Pyelonephritis and Tuberculosis (P.), 7
Pyloric Stricture, Non-Malignant (P.), 333
Quack Drug Bill, The, 23
Quarantine (C.), 412
Queen Victoria's Commemoration (W.), 335
Queen's Reign, The, 259
Railway Nurseries, 375
Refuse. See Hospital Refuse
Renal Diseases (P.), 7
Renal Medicine (P.), 314
Resurrectionist of Old, The, 193
Rheumatism. See p. 69
Rheumatism, Acute (P.), 62
Rheumatism and Gout (P.), 62
Rheumatoid Arthritis (P.), 63
Rhinitis, Pseudo-membranous (P.), 234
Richard III.: A Medico-Psychological Study, 225
Richardson, The Late Sir W. B., 140
Rickets, Nervous Symptoms in (P.), 349
Ringworm: Its Nature, Diagnosis, and Treatment (C.), 247, 263
Roman Influence, The, 43
Röntgen Rays and various Forms of Talipes (P.), 367
Royal Medical and Chirurgical Society (C.), 108, 178, 263, 296, 330, 395
Royal Statistical Society (C.), 362
Saint Thomas's Hospital (A.H.), 36
Sanitation in Health Resorts, 359
Sarcoma and Tuberculous Disease of the Nasal Passages (P.), 31
Scarlatina (P.), 396
Scarlet Fever, The Infection of (C.), 26
Scarlet Fever, Prognosis in, 163
Scarlet Fever Patients, The Isolation of, 358
School Girls, The Health of, 338
School Life, The Regimen of Early, 57
Schools and Diphtheria, 425
Science and the Fragrant Weed, 293
Sclerosis, Amyotrophic Lateral (P.), 164
Sclerosis, Disseminated (P.), 233
Scoliosis, Congenital (P.), 366
Scottish Prisons, The Sick in (W,), 287
Scraps and Gleanings, 36, 68, 100, 118, 136, 155, 187, 205, 222, 240, 255, 290, 354, 385, 403, 438
Septal Deviations (P.), 30
Septum, Deviations, Spurs, and Ridges of the (P.), 299
Serous Expectoration after Paracentesis for Serous Effusion (P.), 130
Serum, Anti-plague, 327
Serum Treatment of Puerperal Fever (C.), 92
Serum Treatment of Diphtheria, 73, 217
Servants and Medical Men, 186
"Shelters" and Sanitation (C.), 379
Shoulder Braces (P.), 350
Shyness, 3
Silver, Two New Salts of (P.), 11
Sinus Empyæmas, Complications of Accessory (P.), 267
Sinus Thrombosis (P.), 149
Sixpenny Doctor, The, 291
Skull, Bone Defects of the (P.), 112
Skull, Surgery of the (P.), 111
Sleeplessness, The Prevalence of, 327
Snake Poison (P.), 398
Sociology. See Modern Sociology
Sore Throat, Membranous (P.), 382
Sound, Subjective Sensation of (C.), 109
Southport, New Infirmary (W.), 99
Spina Bifida and Meningocele (P.), 97
Spinal Caries, Diagnosis of (P.), 98
Spinal Caries, The Treatment of (P.), 98
Spinal Cord, Tumours of the (P.), 98
Spine, Injuries to the (P.), 97
Spine, Neuralgia of the (P.), 97
Spleen, Rupture of the (P.), 316
Spleen, Wandering, The Treatment of (P.), 316
Spleen and Pancreas, Surgery of the (P.), 316
Splenectomy (P.), 316
State Medicine, Progress in (C.), 46
Stenosis, Nasal (P.), 234
Stomach. See *Œsophagus*
Stomach, Carcinoma of the (P.), 199
Stomach, Diseases of the (P.), 282
Street Collections: Hospital Saturday Fund (W.), 135
Stricture, Non-Malignant Pyloric (P.), 333
Student of Medicine, The, 20, 38, 54, 70, 86, 102, 120, 138, 155, 172, 187, 206, 222, 240, 256, 273, 290, 306, 324, 340, 356, 372, 388, 406, 422, 438

Sub-peritoneal Tissue, The (P.), 167
Sugar as Food, 213
Surgery, General, 47, 79
Surgery, Genito-Urinary (P.), 27, 49, 383, 399, 417
Surgery of the Kidneys and Ureters (P.), 432
Surgery of the Liver and Gall Bladder (P.), 284
Surgery of the Nerves (P.), 27, 433
Surgery of the Œsophagus and Stomach (P.), 198, 333
Surgery, Orthopædic (P.), 350, 366
Surgery of the Pancreas and Spleen (P.), 316
Surgery of the Peritoneum (P.), 167
Surgery of the Prostate (P.), 383
Surgery, Respiratory (P.), 129
Surgery of the Skull (P.), 111
Surgery of the Spine (P.), 96
Surgery of the Spleen and Pancreas (P.), 316
Surgery of the Vermiform Appendix (P.), 251
Surgical Ritual, 88
Swansea Hospital. *See p.* 90
Talipes Equino Varus (P.), 367
Tannalbin (P.), 64
Thames and the Sewage, The (W.), 116, 134
Therapeutic Notes *(and sub-titles)* (P.), 11, 50, 63, 132, 200, 217, 252, 334
Therapeutical "Purists," 375
Thiersch's Method (Nerve Surgery) (P.), 29
Thyroid Extract (Treatment of Insanity) (P.), 198
Tic Douloureux (P.), 165, 181
Tilbury Cottage Hospital (W.), 151
Tinea, Circinata, Sycosis (P.), 263
Tongue, Cancer of the (C.), 145
Tongue, Hemiatrophy of the (P.), 248
Torbay Hospital, The, 159
Torticollis, Spasmodic (P.), 249
Tramways and Healthy Dwellings, 224
Transport of Invalids by Rail, The, 72
Tonsils, Tuberculosis of the (P.), 417
Tuberculosis (P.), 7
Tuberculosis, The Extirpation of, 327
Tuberculosis of the Intestine by Ingestion (P.), 349
Tuberculosis, Laryngeal (P.), 416
Tuberculosis of the Nose (P.), 30, 266
Tuberculosis, The Notification of, 408
Tuberculosis Perforans (P.), 112
Tuberculosis, The Prevention and Cure of, 55
Tuberculous Disease of the Hip-joint (C.), 6
Tuberculous Disease of the Nasal Passages (P.), 30, 266
Tuberculous Peritonitis (P.), 167
Tumour of the Face, Congenital (P.), 349
Tumours, Brain (P.), 147
Two Millions for Science, 243
Typhoid Fever (P.), 364
Typhoid Fever, The Diagnosis of, 141
Typhoid, Soldiers', in India, 259
Ulcer, Gastric (P.), 333
Ulcer, Perforating (P.), 216
Ulceration of the Bladder (P.), 49
Union, A Doctors' (W.), 13
Upper Air Passages, Diseases of the (P.), 29, 234, 250, 266, 299
Ureter, Stone Impacted in the (P.), 417
Ureter, Valvular Stricture of the (P.), 418
Ureters, Surgery of the (P.), 432
Urine Analysis (P.), 332
Uterus, Diagnosis of Cancer of the (C. 429
Vaccination. *See p.* 374
Vaccination Crusade, A, 173
Vaccination and Small-pox Statistics. *See p.* 362
Vermiform Appendix (P.), 251
Vertebral Column, Special Affection of the, of Traumatic Origin (P)., 351
Vesical Calculus (P.), 49
Vesical Stone (P.), 400
Wandering Spleen, The Treatment of (P.),
Water Supply (P.), 302
Wells, Death of Sir Spencer, 308
What is a "Medical Charity"? 425
Whitlow (C.), 347
Workshop, The Biggest in the World,
Writers' Diabetes, 292
Writers or Fighters? 409
Xeroform (P.), 50

### THE SPECIAL SUPPLEMENT FOR NURSES.

"Admitted as Urgent," 114
Africa, Notes from South, 100, 234
American Letter, Our, 46, 70, 198, 235
Appointments, 10, 18, 28, 39, 47, 57, 66, 74, 84, 100, 113, 123, 135, 144, 156, 162, 181, 190, 196, 208, 214, 223
Appointments, Minor, 5, 18, 28, 39, 48, 56, 65, 74, 83, 88, 92, 98, 116, 123, 148, 164, 174, 182, 190, 200, 208, 214, 224, 234
Book World for Women and Nurses, The, 57, 136, 164, 173, 191
Book and Its Story, A, 8, 26, 55, 82, 90, 147, 163, 226
"Burdett's Official Nursing Directory," 27
Caged Orphans at Broadstairs, The, 93
Canadian Nurses in the United States, 134
Canary Islands for Health or Pleasure, The, 170
Chelsea Infirmary; Opening of New Nurses' Home, 53
Christmas Books, 109, 115
Christmas Gifts, 108
Christmas Gifts for the Hospitals, Our, 97, 120
Christmas Story, A, 107
Christmas in a Country Workhouse Infirmary, 113
Christmas in the Dublin Hospitals, 104
Christmas in the East-end, 112
Christmas in Egypt, 107
Christmas in the Fatherland, 106
Christmas at the Hospitals, 123, 129
Christmas at Marseilles, 106
Christmas in Military Hospitals in India, 104
Christmas in the United States, My First, 105
Christmas with the Women Students, 115
City of London Union Infirmary, 27
Club for Nurses, A, 169
Colonial Nursing Association, The, 214
Conference of Women Workers at Manchester, 52
Cookery Classes at the London Hospital, 72
Council of County Nursing Association, 73
Death in Our Ranks, 24, 46, 53, 76, 144, 152, 215, 219
Dress and Uniforms, 9, 133, 171
East London Nursing Society, 205
Everybody's Opinion, 11, 19, 27, 40, 47, 56, 65, 75, 83, 94, 101, 109, 123, 135, 145, 155, 161, 172, 180, 189, 199, 207, 215, 227, 235
Female Sanitary Inspectors, 11
Gloucester District Nursing Society, 92
Hall Caine, Mr., at sea, 154
Help the Nurses to Help the Sick, 110
Hints to Nurses Going to India, 4
Hints to Nurses Going to the Riviera, 64
Hospital Entertainments, 145
How Nurses can Commemorate the Year 1897, 196
Humane Influence of the Modern Hospital, The, 129
Hygiene for Nurses, 3, 15, 23, 31, 43, 51, 61, 69, 79, 87, 97, 119, 127, 139, 151
Johannesburg Hospital, The, 144
Johannesburg Hospital and the Nurses, The, 215
Lady Dufferin's Fund, 120
Lectures to Surgical Nurses, 185, 203, 219
Legal Intelligence, 121
Lockers and Larders, 57
Mediæval Practice in the Care of the Sick, 10, 32, 62
Midwifery Papers, 6, 16, 34, 72, 80, 91, 153, 160
Midwives' Institute and Trained Nures' Club, 162
Midwives' Registration Bill, 1897, The, 188
Midwives, Registration of, 235
News from the Nursing World, 1, 13, 21, 29, 41, 49, 59, 67, 77, 85, 95, 103, 111, 117, 125, 137, 149, 157, 165, 175, 183, 193, 201, 209, 217, 229
Notes and Queries, 12, 20, 28, 40, 48, 58, 66, 76, 84, 94, 102, 116, 124, 136, 148, 156, 164, 174, 182, 192, 200, 208, 216, 228, 236
Novelties for Nurses, 7, 39, 57, 66, 84, 98, 110, 113, 156, 233
Nurses' Association, Quarterly Council Meeting of the Royal British, 141
Nurses' Association, Royal British, 35, 46, 62, 113
Nurses' Association, The British, 18
Nurses' Clinic, Trained, 24, 44
Nurses' Club, Trained, 92
Nurses in 1896—Their Quarters, Hours, and Food, 5, 17, 25, 33, 45, 54, 63, 71, 81, 89, 99
Nurse's Friend, A, 7
Nurse's Visit to Texas, A, 140
Nursing in America, 19, 223
Nursing in Australia, Private, 83
Nursing in the Colonies, 65
Nursing in India, 179
Nursing in L.C.C. Asylums, 76
Nursing at the London Hospital, 206
Nursing in New South Wales, 179
Nursing Order, A New Military, 205
Persistent Libel, A, 143
Pharmacy and Dispensing for Nurses, 59, 167, 177, 195, 211, 231
Pirated Advertisements, 101, 115, 134
Plague, Nurses for the, 206, 213
Poor Law Nurses and the Superannuation Act, 52
Poor Law Officers' Superannuation Act, The, 38
Post-Graduate Clinics for Nurses, 168, 178, 186, 204, 212, 220, 232
Practical Hints for New Probationers, A Few, 169
Practical Points, 206
Presentations, 32, 54, 83, 122, 135, 140, 182, 204, 223, 233
Prince Alfred Hospital, Sydney, 188
Queen Victoria's Jubilee Institute for Nurses, 120
R.B.N.A. "Irreconcilables" and Mental Nurses, 141
Reading to the Sick, For, 12, 20, 48, 58, 76, 84, 102, 116, 124, 136, 148, 156, 174, 192, 216, 228
Real Nurses, 225
Royal National Pension Fund for Nurses, The, 128, 221, 233
Royal Visit, A, 224
St. Bartholomew's Nurses in Danger, 83
St. Luke's Home, Vancouver, 225
Seven Weeks in a London Fever Hospital, 187, 197
Sketches of Hospital Life, 98
Very "Peculiar" Home of Rest, A, 213
Victoria Club for Nurses, The, 180
Victoria Commemoration Club, 233
Wants and Workers, 27, 65, 82, 155, 182, 226, 236
What Doctors expect from Nurses, 88
Where to Go, 17, 38, 46, 58, 66, 75, 81, 94, 100, 124, 134, 144, 154, 164, 169, 182, 191, 200, 208, 213, 225, 236
Workhouse Infirmary Nursing Association, 214

Printed by W. H. and L. Collingridge, "City Press," 148 and 149, Aldersgate Street, London, E.C.

### EARLY EDITION.

**Notices of Births, Deaths, and Marriages** are inserted in this column at a charge of 2s. 6d. for an announcement not exceeding four lines.

### NOTICE TO CORRESPONDENTS.

All MS., Letters, Books for Review, and other matters intended for the Editor should be addressed The Editor, The Lodge, Porchester Square, London, W.

The Editor cannot undertake to return rejected MS., even when accompanied by stamped directed envelope.

**Notice to Advertisers and Subscribers.**

All Advertisements, Orders for Copies of The Hospital, and Business Communications should be addressed to **THE MANAGER, (Not to The Editor),** The Hospital, 28 & 29, Southampton Street, Strand, London, W.C.

The Cost of Subscription, post free, is as follows:—Per week, 2½d.; per quarter, 2s. 8d.; per half-year, 5s. 3d.; per annum, 10s. 6d. Foreign:—Per annum, 15s. 2d.; payable, in all cases, in advance.

**IMPORTANT NOTICE.**

On and after October 1st, 1896, the EDITORIAL and PUBLISHING OFFICES of "THE HOSPITAL" will be TRANSFERRED to their new premises, 28 & 29, SOUTHAMPTON STREET, STRAND, LONDON, W.C.

**NOTICE.—Vol. XX. of "The Hospital" (April to September, 1896), in handsome cloth binding, will be on sale at the Office of this Journal, early in October, price 7s. 6d. Cases for binding the Numbers contained in this Volume 1s. 6d. Index to Vol. XX., 2½d., post free.**

**The Monthly Part for October is now ready, Price 6d., by post 8d.**

### NOTES AND NEWS.

### The Student of Medicine.

### APPOINTMENTS.

S. J. Aarons, M.B., C.M., appointed Resident Medical Officer to the Royal Edinburgh Hospital for Sick Children. H. B. Balfour, M.B., C.M., appointed Resident Medical Officer to the Royal Edinburgh Hospital for Sick Children. Astley V. Clarke, M.B., B.C., B.A.Cantab., M.R.C.S., L.R.C.P., appointed Honorary Assistant Physician to the Leicester Infirmary. A. Vernon Davies, M.B., Ch.B.Vict., appointed House Surgeon to the Clinical Hospital, Manchester. Henry Duffus, M.A., M.B., C.M., appointed Medical Officer for the Parishes of Marykirk and Logie-Pert. Dr. Duncan, appointed Senior House Surgeon to the Cumberland Infirmary, Carlisle. John C. Hackett, M.D.R.U.I., M.Ch., appointed Medical Officer to the Dungarvan Union. E. Hilliard, L.R.C.P., L.R.C.S.Edin., appointed Medical Officer to the Blackpool Post Office. Thomas Jones, M.R.C.S.Eng., L.R.C.P.Lond, L.S.A., appointed Senior House Surgeon to the Bootle Borough Hospital. B. H. Lees, M.A., M.B., B.C.Camb., appointed Medical Officer for the No. 2 B (Worthing) District of the East Preston Union. John D. S. Nodes, L.R.C.P.Lond, M.R.C.S.Eng., appointed Public Vaccinator for the No. 2 B (Worthing) District of the East Preston Union. Thomas Pickering Pick, F.R.C.S., appointed Inspector of Anatomy for the Provinces. H. L. Thurnell, M.A.Cantab., M.R.C.S.Eng., L.R.C.P. and L.S.A.Lond., appointed Surgeon to Her Majesty's Customs at Gravesend. W. H. Vickery, F.R.C.S.Eng., appointed Honorary Surgeon to the Hospital for Sick Children, Newcastle-on-Tyne. Ernest E. Ware, M.D., M.R.C.S., etc., elected on the Acting Medical Staff of the Hampstead Hospital. Samuel Wilks, M.D., F.R.S., President of the Royal College of Physicians, appointed Consulting Physician to the Hampstead Hospital. A. W. Woodroffe, M.D.Dub., L.R.C.S.I., appointed Medical Officer for the Workhouse and for the Ticehurst District of the Ticehurst Union.

### University of Durham.

Second Examination for the Degree of Bachelor in Medicine. The following candidates have satisfied the examiners:—

Anatomy, Physiology, Materia Medica.—Honours, Second Class: E. Gofton, College of Medicine, Newcastle-upon-Tyne; J. Muirhead, College of Medicine, Newcastle-upon-Tyne; N. C. Bailes, College of Medicine, Newcastle-upon-Tyne; W. Sisam, Mason College, Birmingham; J. W. H. Boyd, College of Medicine, Newcastle-upon-Tyne. Pass List: S. A. Arthur, College of Medicine, Newcastle-upon-Tyne; A. Ayre-Smith, Guy's Hospital; C. O. Bodman, University College, Bristol; F. W. Burn, College of Medicine, Newcastle-upon-Tyne; C. H. Brookes, College of Medicine, Newcastle-upon-Tyne; F. A. Davies, Mason College, Birmingham; H. B. Fawcus, College of Medicine, Newcastle-u on-Tyne; J. R. Halliday, College of Medicine, Newcastle-upon-Tyne; J. A. Hartington, College of Medicine, Newcastle-upon-Tyne; A. A. Hope, College of Medicine, Newcastle-upon-Tyne; W. J. Harrison, College of Medicine, Newcastle-upon-Tyne; J. Harris, B.A., Sydney University; J. T. Johnson, College of Medicine, Newcastle-upon-Tyne; J. Milligan, College of Medicine, Newcastle-upon-Tyne; G. B. Picton, College of Medicine, Newcastle-upon-Tyne; N. Roberts, College of Medicine, Newcastle-upon-Tyne; Grace Harwood Stewart, London School of Medicine for Women; W. J. Symes, Sheffield School of Medicine; S. Southam, Owens College, Manchester; A. W. Tuxford, St. Mary's Hospital; H. B. Thompson, Mason College, Birmingham; R. H. Vincent, St. Bartholomew's Hospital; C. W. Von Bergen, College of Medicine, Newcastle-upon-Tyne.

Mr. S. C. Vincent, M.B., B.S.Durh., has passed the examination for Bachelor in Hygiene; and Mr. E. Percy, M.B., B.S.Durh., that of Diploma in Public Health.

### ROYAL NATIONAL PENSION FUND FOR NURSES.

*President*—H.R.H. THE PRINCESS OF WALES.

*Chairman*—WALTER H. BURNS, Esq. *Deputy-Chairman*—HENRY C. BURDETT, Esq.

PENSIONS. SICKNESS. ACCIDENT.

### Total Invested Funds, £300,000.

Nurses are invited to join the Fund on account of the substantial and exceptional advantages which it offers them and which *they cannot obtain elsewhere.* The following are the chief points:—

1. **The Fund is Mutual and essentially Co-operative.**
2. **Easy Payment of Premiums.**
   Nurses can pay their premiums monthly or otherwise as best suits their convenience
3. **The Fund is open to every Nurse.**
   Nurses can assure for Pensions of any amount commencing at any age.
4. **An Investment and Savings Bank.**
   Those entering under the returnable scale can have their premiums returned to them with compound interest, less a small deduction for working expenses. Interest allowed on deposits.
5. **Additions to Pensions.**
   Besides the Pension entered for, substantial additions thereto may be anticipated in the shape of bonuses.
6. **Sickness and Accident Assurance.**
   Policies are issued in connection with Pension policies assuring 10s., 15s., or 20s. a week in cases of incapacity from work through sickness or accident.

**Full particulars to be obtained from THE SECRETARY,**
ROYAL NATIONAL PENSION FUND FOR NURSES,
28, FINSBURY PAVEMENT, LONDON, E.C.

